# Functional Near-Infrared Spectroscopy in neurodegenerative disease: a review

**DOI:** 10.3389/fnins.2024.1469903

**Published:** 2024-10-02

**Authors:** Lei Xie, Yong Liu, Yuling Gao, Jiaqi Zhou

**Affiliations:** The First Affiliated Hospital of Dalian Medical University, Dalian, China

**Keywords:** Functional Near Infrared Spectroscopy (fNIRS), neurodegenerative diseases, neurovascular coupling, Parkinson, Alzheimer disease, multi-modality imaging

## Abstract

In recent years, with the aggravation of aging, the incidence of neurodegenerative diseases is increasing year by year, and the prognosis of patients is poor. Functional Near-Infrared Spectroscopy (fNIRS) is a new and non-invasive neuroimaging technology, which has been gradually deepened in the application research of neurodegenerative diseases by virtue of its unique neurooxygen signal brain functional imaging characteristics in monitoring the disease condition, making treatment plans and evaluating the treatment effect. In this paper, the mechanism of action and technical characteristics of fNIRS are briefly introduced, and the application research of fNIRS in different neurodegenerative diseases is summarized in order to provide new ideas for future related research and clinical application.

## 1 Introduction

Neurodegenerative diseases (Pasko et al., 2022) (NDDs) are a group of diseases characterized by cognitive and motor deficits in patients due to progressive degeneration of neurons in the brain or spinal cord and/or degeneration of myelin sheath function, including Parkinson’s disease, Alzheimer’s disease, Huntington’s disease, amyotrophic lateral sclerosis, and multiple systemic atrophy. The risk of NDDs increases with the increase of age, especially in the elderly. At present, there is no effective radical cure. In recent years, the incidence of NDDs has shown an increasing trend from year to year as aging increases (Logroscino et al., 2022). Previously, the diagnosis and assessment of NDDs relied mainly on clinical manifestations and traditional imaging, lacking direct probes using cerebral blood oxygen metabolism as an indicator. Functional Near-Infrared Spectroscopy (Rahman et al., 2020; Curtin et al., 2019) (fNIRS) is an emerging, non-invasive optical imaging technique in recent years, which can reflect the level of brain activity by detecting changes in parameters such as oxyhaemoglobin in brain tissue. The fNIRS technique acquires cerebral blood oxygenation signals and then combines them with signal analysis methods to extract effective metric parameters such as brain activation and brain network connectivity, which can be used as biological indicators for the diagnosis and evaluation of NDDs. In addition, fNIRS can provide treatment-related information on cortical activation and changes in brain network connectivity, which has important clinical applications in assessing the extent of disease in NDDs, guiding treatment, and evaluating efficacy. In some functional areas related to social cognition, language control and thought computing, such as prefrontal cortex (PFC) and inferior parietal lobule, fNIRS can effectively detect this area. Measuring PFC activation in patients with NDDs versus healthy controls using fNIRS can help in the diagnosis and disease assessment of patients with NDDs. This paper summarizes the basic principle of fNIRS technology and its application research progress in NDDs in recent years, analyzes the advantages and disadvantages of this technology and puts forward the prospect in the future.

## 2 Fundamentals of fNIRS

The fNIRS technology is based on the optical properties of biological tissues and takes advantage of the good penetration ability of NIR light into biological tissues. The light source emits NIR light to a specific brain region, and the NIR light emitted after a series of absorption and propagation in the biological tissues carries biochemical information related to the optical properties of the tissues. The dominant and physiologically dependent absorbing chromophores in biological tissues are oxygenated hemoglobin (HbO) and deoxygenated hemoglobin (HbR), and there is a significant difference in near-infrared (NIR) light absorption in the 650–950 nm band between the two chromophores. The main purpose of fNIRS is to quantitatively measure the concentration of two kinds of absorption chromophore in tissue, and to provide convenient and reliable monitoring index for clinical and scientific research by obtaining useful information related to tissue structure and physiological function.

There are about 12–14 billion neuron cells in the human brain, which have the function of communicating and integrating input information and outputting processed information. When neuron cells are resting, HbO in the blood releases oxygen at a certain rate and converts it into HbR. The ratio of HbO to HbR remains relatively stable. The oxygen released during this process is absorbed by neurons for basic metabolic activities. When neuron cells are stimulated and activated, their demand and consumption for oxygen will greatly increase, resulting in a rapid decrease in the concentration of HbO and an increase in HbR. Changes in the concentration of HbO and HbR in the blood will cause cerebral blood vessels such as the arteriolar sphincter to relax (Iadecola, 2017), and the corresponding regional cerebral blood flow (CBF) and cerebral blood volume (CBV) increase to provide more oxygen-rich fresh blood (Nippert et al., 2018). During this process, the amount of oxygen replenished will be much more than the actual consumption of neurons. This produces an “Overabundance” effect, which ultimately causes the concentration of HbO in the blood of the brain to increase and the concentration of HbR to decrease accordingly. This change in blood oxygen level caused by neural activity is called Neurovascular Coupling (Irani et al., 2007; Scholkmann et al., 2014). This mechanism is the basis of fNIRS imaging. fNIRS does not directly measure the activity of neurons themselves, but monitors the physiological changes caused by the activity of neurons in the brain, namely changes in HbO and HbR concentrations, and reflects the activity of neurons in the brain.

NIR is an electromagnetic wave between visible light (VIS) and mid-infrared light (MIR), with a wavelength of 780–2526 nm. The absorption coefficient of biological tissue changes with the change of wavelength. In the 650–950 nm band, NIR has a strong ability to penetrate biological tissue. This band is also called the optical window of biological tissue (Strangman et al., 2002). By placing the NIR light source on the surface of the scalp, photons will enter the brain tissue through the scalp and skull. After being absorbed and scattered by the brain tissue, they will return to the surface of the scalp and be detected by the detector. HbO and HbR absorb NIR light differently: HbR absorbs stronger below 800 nm, and HbO absorbs stronger above 800 nm. This absorption difference is also reflected in the blood color, namely arterial blood (≈98% saturated) is redder, and venous blood (≈75% saturated) is purple-colored (Pinti et al., 2020). Select NIR with one wavelength on both sides of 800 nm, and detect the change in light intensity when it passes through the brain. Compare the light intensity during the stimulus presentation period with that during the non-stimulus and baseline period, and infer that the two absorbing light groups, HbO and HbR, can indirectly obtain information on neural activity (Ferrari and Quaresima, 2012). Since the first non-invasive measurement of changes in hemoglobin concentration in human brain tissue using the fNIRS technique by Jöbsis (1977), fNIRS is nowadays a promising, safe and non-invasive emerging research method in neurorehabilitation for neuroimaging.

## 3 Strengths and weaknesses of fNIRS

fNIRS has its unique advantages over commonly used functional brain imaging techniques. Compared with common neuroimaging techniques such as functional magnetic resonance imaging (fMRI), electroencephalography (EEG) and positron emission tomography (PET), fNIRS has the strengths of simplicity of operation, low cost, safety, non-invasiveness, compatibility, and higher temporal resolution (Lim et al., 2022). Currently, fNIRS is also the only neuroimaging technique that can accurately detect concentrations and changes in HbO, HbR, total hemoglobin (HbT), and CSF at the same time, enabling effective studies of the temporal dynamics and stability of hydrodynamic coupling. At the same time, there are also relative shortcomings.

fNIRS can be used in combination with other techniques to achieve complementary properties. For example, in Parkinson’s disease, fNIRS can be used to detect the therapeutic effects of transcranial direct current stimulation (tDCS) to improve postural balance and mobility in Parkinson’s disease patients. For Parkinson’s disease and amyotrophic lateral sclerosis, hybrid EEG-fNIRS modeling enhances the diagnosis and assessment of the disease. fNIRS also has some weaknesses, fNIRS can only detect the level of blood oxygen metabolism in the superficial layer of the brain, the ability of localization is limited, and the signal range can not cover the whole brain.

### 3.1 Strengths of fNIRS

When it comes to motion artifacts, fNIRS outperforms other neuroimaging techniques. When using fMRI to monitor infants, infants need to be sedated or asleep, otherwise motion artifacts may appear during the measurement (Fransson et al., 2009). fNIRS, as one of the standard care tools in pediatric icu, does not have this limitation and can directly and real-time evaluate the local oxygenation of infants and young children in conscious states (Balakrishnan et al., 2018; Hoffman et al., 2017). fNIRS is not limited by posture, and can observe the activation of the cerebral cortex in the upright state and the task state (Noah et al., 2015). In terms of temporal resolution, fNIRS has better temporal resolution than fMRI and PET: fNIRS (Jasdzewski et al., 2003) can sample and observe brain signals with a temporal resolution of 0.01 s, and can directly and quickly measure neuronal signals for timely hemodynamic analysis. The comparison of four neuroimaging techniques, fNIRS, fMRI, EEG and PET, is shown in Table 1.

**Table 1:** Comparison of four neuroimaging techniques: fNIRS, fMRI, EEG and PET.

	Temporal resolution	Spatial resolution	Cost	Affected by artifacts	Hemodynamics	Penetration depth	Applicable population	Convenience
fNIRS	Around 10 Hz	Low	Low	Low	HbO, HbR	Brain cortex	Enormous	High
fMRI	1-3 Hz	Extremely high	High	High	HbR	Entire head	Limited	Low
EEG	256–1,024 Hz	Low	Low	High	None	Brain cortex	Enormous	High
PET	Extremely Low	High	High	High	None	Entire head	Limited	Low

### 3.2 Weaknesses of fNIRS

As a reliable method of studying the function and structure of the cerebral cortex, fNIRS provides detailed information that helps understand neural activity patterns in specific brain areas. By observing the activation status of damaged brain areas under different states, the compensation patterns of healthy brain areas, and the differences in functional connections between brain areas, it is helpful to the diagnosis and prognosis of the disease. However, fNIRS still has many shortcomings that limit its further development in cognitive neurology and clinical practice. First of all, the biggest shortcoming of fNIRS is that its maximum detection distance is only about 3 cm below the skull, which limits it to be able to only detect changes in HbO and HbR in specific cortical areas such as PFC (Keles et al., 2016), but is powerless when detecting deep brain areas such as basal ganglia. The detection of fNIRS requires good contact between the probe and the scalp, so it is inevitable that the probe signal will be affected by thick and excessively dark hair or skin color, resulting in the failure of good coupling between the photopole and the scalp. At the same time, if a shift in the probe position occurs during the detection process, although it is less affected by motion artifacts than PET and EEG, it will inevitably lead to relative errors in signal reception (San Juan et al., 2017). These factors are the causes of errors in the detection data and reduce its resolution. At the same time, like most non-invasive neuroimaging modalities, raw fNIRS signals often contain mixed physiological signals or other signals originating outside the cerebral cortex. Ignoring confounding physiological signals may lead to false positives, i.e., the detected hemodynamic changes are mistakenly regarded as functional brain activity; or false negatives occur, and a true cerebral functional hemodynamic response cannot be observed. Data analysis needs to be used to remove misleading physiological signal noise to improve cortical sensitivity.

### 3.3 Application of multimodal imaging

At present, the application of multimodal imaging has become the main driving force in promoting the growth of neuroimaging research.

Multimodal functional neuroimaging leverages the characteristics of complementary different types of neuroimaging techniques, such as the fMRI-EEG/MEG and EEG-fNIRS hybrid models. Many multimodal functional neuroimaging studies combine methods of measuring electrical signals related to brain neurons with hemodynamic measurements to conduct more comprehensive brain science research. In recent years, the EEG-fNIRS model has gradually been used in brain science research (Uchitel et al., 2021).

For movement disorders, studying the coupling between brain and muscle contraction is of great benefit to disease research. For example, when studying Parkinson’s disease, focus on exercise tasks such as muscle contraction or finger flexion and extension. EEG or MEG (Magnetography) can be used as a reliable neurofunctional imaging technology for monitoring exercise tasks (Zheng et al., 2016; Xu et al., 2017). However, these techniques are prone to motion artifacts, which affects their testing on large-scale exercise tasks. fNIRS is not easy to produce motion artifacts. Interestingly, the low temporal resolution of fNIRS can just be compensated for by the high temporal resolution of EEG. Therefore, the research on the application of the hybrid EEG-fNIRS model to Parkinson’s disease has gradually increased in recent years. Abtahi et al. (2020) used the hybrid EEG-fNIRS model to monitor and capture physical movement tasks, and successfully distinguished Parkinson’s disease from neurotypical, which is of certain significance for the early diagnosis of Parkinson’s disease.

## 4 Application of fNIRS in neurodegenerative diseases

### 4.1 Parkinson’s disease

Parkinson’s disease (Morris et al., 2024) (PD) is a progressive neurodegenerative disease commonly seen in middle-aged and elderly people, with the main pathological features of deformed necrosis of nigral dopaminergic neurons and accumulation of residual intraneuronal alpha synaptic nucleoprotein into Lewy bodies. The main clinical manifestations of PD include postural, gait, and cognitive abnormalities, resting tremor, and bradykinesia, among other clinical manifestations. Since patients with PD are often associated with typical motor deficits, making it difficult to lie still and flat or perform some motor tasks, and conventional imaging tests such as fMRI cannot assist in the diagnosis, the clinical diagnosis of PD relies mainly on the clinical manifestations. The fNIRS provides an auxiliary diagnostic tool for the clinic due to its good motor anti-interference property, which can intuitively reflect the cerebral cortical activation of PD patients under motor tasks.

Dopamine is an important neurotransmitter, and diminished PFC activity in PD patients is associated with reduced dopamine levels. The PFC is a joint area involved in the integration of some motor functions and sensations, as well as being the site of executive attention, initiating purposeful and goal-oriented behaviors essential for daily life (Criado et al., 1997). The PFC is associated with executive function, and it performs relevant tasks through cortical-striatal dopaminergic pathways in different regions. Executive function is an umbrella term encompassing a wide range of cognitive abilities, including problem solving, rule switching, task switching, working memory manipulation, and response inhibition. The left prefrontal lobe is associated with learning new motor sequences or with improving motor performance through repetitive practice, and the right prefrontal lobe plays a dominant role in maintaining, controlling, and executing movement changes, both of which are necessary functions during dual-tasking and walking across obstacles.

#### 4.1.1 Application of fNIRS in PD gait abnormalities

Gait abnormality (Mirelman et al., 2019) (PIGD) is not only a typical feature of PD, but also an important cause of disability in the later stage of the disease, which is characterized by the decrease or disappearance of arm swing of the affected upper limb and drag of the lower limbs. A study (Koenraadt et al., 2014) measured haemodynamic changes in PFC in healthy individuals using fNIRS. 11 subjects were randomized to 10 3 km/h treadmill walking trials (normal walking) and 10 3 km/h treadmill walking trials (precision stepping). The walking test and rest time were carried out alternately. Before both walking trials, the PFC showed profound activation, as evidenced by increased HbO and decreased HbR. Compared with normal walking, the precise step task showed more PFC activation in the first half of the task. The study confirms that PFC is activated during tasks requiring concentration and that fNIRS is suitable for recording the planning and initiation process of gait. Hamacher et al. (2015) provided the first systematic summary of the current state of research on brain activity during gait tasks using EEG, fMRI and fNIRS, emphasizing the importance of PFC control in gait as well as the need to apply fNIRS to gait disorders. Maidan et al. (2016) founds that there are significant differences in the impact of walking conditions on HbO levels. In healthy elderly people, compared with usual walking, HbO increased during dual-task walking, and a significant increase was observed during the process of avoiding obstacles. Trend of change. PD patients need to avoid obstacles when walking in complex environments, which requires the use of additional cognitive resources such as distraction and visual scanning to allocate motor resources. During dual task, compared with usual walking, no significant changes in HbO levels were observed during dual task walking, but increased during obstacle avoidance, indicating that PFC activation depends on the nature of the task. These findings may have implications for PD patients. Gait rehabilitation is of great significance. Commonly used drugs in PD treatment include dopaminergic and cholinergic drugs, which can improve the gait of PD patients during complex walking and promote HbO recruitment in the PFC (Zhou et al., 2023). In 2021, a randomized controlled trial (Orcioli-Silva et al., 2021) recruited 23 PD patients and 30 healthy elderly people. In a trial of barrier-free walking and obstacle avoidance, patients with PD were treated with and without medication. Near-infrared spectroscopy was used to measure HbO concentration and PFC activation in healthy controls and PD groups. The results of the study showed that compared with walking without obstacles, PD patients taking medication had increased PFC activation during obstacle avoidance. In contrast, PFC activation was reduced during obstacle avoidance compared with obstacle-free walking in the unmedicated state. The PFC activity of the healthy control group was higher when there were obstacles than when walking without obstacles. This indicates that PD patients in the unmedicated state exhibit a compensatory mechanism and PFC activity does not increase during the disorder, indicating that drugs can improve the gait of PD patients during complex walking.

#### 4.1.2 Application of fNIRS in PD postural abnormalities

Postural abnormalities are another typical feature of PD, characterized by trunk tilt, spinal deformity and balance abnormalities. The PFC has an important role in postural control. A randomized controlled trial (Mahoney et al., 2016) compared neural activation patterns, postural stability and their interactions in prefrontal cortex of PD patients and healthy controls using fNIRS as a cortical probe. The study included 26 patients with PD, 117 patients with mild signs of Parkinson’s disease and 126 healthy controls. Participants were asked to stand upright and be silent for 10 s while changes in prefrontal cortex oxyhaemoglobin levels were measured using fNIRS. The results of the study suggest that people with PD require more prefrontal activation to maintain an upright posture compared to healthy older adults. This study highlights the role of the PFC in postural control in PD patients and provides an opportunity to improve treatment options for postural instability.

#### 4.1.3 Application of fNIRS in PD cognitive impairment

Parkinson’s disease with cognitive impairment (PDCI) is one of the common and important non-motor symptoms of PD, and the main cognitive domains that are impaired are attention, executive function, episode learning and memory, and visuospatial abilities. Studies have shown that nearly 50% of cognitively normal PD patients progressively experience cognitive decline after 6 years, while patients diagnosed with PD for more than 10 years have further cognitive impairment and eventually develop Parkinson’s disease dementia. The Drawing the Clock Test (CDT) (Jalakas et al., 2019) is a neuropsychological test used to screen overall cognitive function. The test requires the use of several cognitive domains including executive function, visuospatial ability, and semantic memory, and can be a suitable tool to screen for cognitive decline in patients with early stage PD. fNIRS assesses neural activation during real-time performance of the CDT. Schejter-Margalit et al. (2022) included 29 patients with PD and 31 healthy controls and combined the fNIRS for CDT measurement of neural activation in the PFC. The study found that the HbO increase slope of PD patients when performing tasks was lower than that of normal people, that is, the PFC activation was slower than that of healthy controls, showing a decline in cognitive ability. It also shows the importance of performing CDT to detect early cognitive decline in PD patients.

#### 4.1.4 Multimodal research applied to PD

tDCS (Weller et al., 2020) can increase PFC excitability and improve PFC cognitive function and gait. fNIRS can be used to evaluate the hemodynamic response related to tDCS treatment (Patel et al., 2020). In 2021, a randomized controlled trial (Conceição et al., 2021) investigated the effect of adding tDCS to PFC on gait, cognition and PFC activity while walking during aerobic exercise in PD patients. Twenty PD patients were included who participated in two 30-min aerobic exercise sessions and received different tDCS conditions (2mAtDCS or sham control) for 1 week, with the treatment target PFC in the most affected cerebral hemisphere. The study found that compared with before intervention, aerobic exercise +tDCS stimulation increased HbO levels and decreased HbR levels in the stimulated hemisphere, while no significant changes were observed in aerobic exercise + sham tDCS. Significant changes were observed in both aerobic exercise +tDCS stimulation and aerobic exercise + sham tDCS stimulation in the non-stimulated hemisphere. This suggests that walking reduces steptime variability in the stimulated hemisphere, is manifested by faster responses, and increases PFC activity (increased HbO levels and decreased HbR levels), which were not observed in aerobic exercise alone. Research has shown that the combination of aerobic exercise and tDCS is expected to be a way to enhance gait intervention. The study used fNIRS as a Detection Tools to demonstrate that adding tDCS to aerobic exercise has an immediate positive impact on gait variability, processing speed, and execution control in PD patients.

Deep brain stimulation (Vitek and Starr, 2020) (DBS) surgery is one of the most important treatments for PD. Due to metal artifacts and safety concerns, patients undergoing DBS surgery should be used with caution when undergoing MRI. fNIRS (de Hemptinne et al., 2015) does not measure activity in deep brain regions such as the basal ganglia, but it is able to safely measure cortical activity in patients with DBS to assess the therapeutic effects of DBS surgery. Yu et al. (2021) recruited five patients with PD who underwent DBS surgery, and the patients performed a different DBS frequencies to complete a 10-meter walking task and wore an fNIRS system to measure changes in the patients’ prefrontal HbO concentrations. The study presents the first fNIRS-based assessment and optimisation of DBS treatment regimens, which is expected to provide an objective solution for patient-specific optimisation of DBS treatment.

#### 4.1.5 Summary

Parkinson’s disease is one of the most studied neurodegenerative diseases applied by fNIRS. fNIRS, as a unique neuroimaging technology, has valuable potential application value in early detection of changes and differences in PFC activation, and detection of subtle changes in brain activity of PD participants and healthy controls in the relatively early stage of the disease (Schejter-Margalit et al., 2022), which helps detect early cognitive decline in PD patients and is of great significance for improving patient prognosis and delaying disease progression. The study of fNIRS in PD may provide a theoretical basis for targeting neuromodulation techniques. At the same time, fNIRS has good compatibility. Multimodal neurological functional imaging combined with EEG and other technologies can further expand the application of fNIRS in clinical treatment and provide more complete brain functional information. However, the application of fNIRS in the field of PD is still in its infancy and has certain limitations. fNIRS cannot be directly used as a diagnostic method for PD. It is an auxiliary diagnostic method that indirectly reflects brain dysfunction by monitoring brain hemodynamics. The imaging depth of fNIRS is mainly limited to PFC, but has not been fully studied in other cortical areas, and the changes in subcortical structures under different exercise task conditions are still unknown. In addition, the current relevant research measurement indicators and research methods are single, and there is a lack of discussion on the connection and integrity between limb movements and brain areas. There is still a need for more samples and higher-quality fNIRS research to further clarify various neural structures and provide new ideas for PD diagnosis.

### 4.2 Alzheimer’s disease

Alzheimer’s disease (AD) is the most common age-related neurodegenerative disease, its etiology and pathogenesis are not completely clear, the current characteristic pathological changes are abnormal *β*-amyloid protein deposition in the brain. It occurs in old age, accompanied by progressive behavior and cognitive impairment (Scheltens et al., 2021). AD is one of the major causes of dementia and seriously affects the prognosis of patients. Globally, it is estimated that 32 million, 69 million and 315 million people suffer from AD dementia, prodromal AD and preclinical AD, respectively. They totalled 416 million people in the AD continuum, representing 22% of the global population of 1.9 billion people aged 50 years and older (Gustavsson et al., 2023).

#### 4.2.1 Haemodynamics in Alzheimer’s disease

Currently, the diagnosis of AD relies on patient history and clinical scores such as the Brief Mental Status Examination (MMSE) scale or the Clinical Dementia Rating Scale (CDR). In recent years, brain imaging techniques such as fMRI and PET have been used intensively in AD. fMRI visualizes AD-related cortical atrophy and captures changes in brain connectivity. On this basis, fNIRS provides functional imaging by measuring cortical haemodynamic responses making fNIRS a potential alternative to fMRI and PET for more convenient and non-invasive clinical diagnosis or monitoring of AD patients. As early as 1996 Hock et al. used fNIRS to monitor AD haemodynamic responses. This study found (Hock et al., 1996) that in young healthy subjects, frontal cortical HbO increased and HbR decreased during the performance of a computational task; in older healthy subjects, mean HbO and HbR were significantly lower than normal during the computational task, followed by a significant increase in the concentrations of HbO and HbT during brain activation; and in comparison to older healthy subjects, AD patients performing a computational task had significantly lower HbO and HbT levels were significantly lower compared to baseline. This phenomenon may be due to a significant reduction in local cerebral blood flow over degenerating brain regions such as the parietal cortex during functional brain activation and is related to the development and time course of neurodegeneration. A clinical trial (Ferdinando et al., 2023) recruited 8 patients with AD and 14 healthy controls, and all participants were subjected to MMSE scoring and 5-min fNIRS testing. The results of the study showed that the MMSE scores of AD patients were mildly demented compared to healthy controls. In the early stages of AD, vascular-driven cerebrospinal fluid transport is reduced, and reduced haemodynamics in AD patients is associated with a lag in cerebrovascular-cerebrospinal fluid coupling, which is visualized on the MMSE score.

#### 4.2.2 fNIRS and mild cognitive impairment

Mild cognitive impairment (MCI) (Rabin et al., 2017) is one of the main manifestations of AD, and early diagnosis of MCI can help prevent the development of cognitive impairment. A randomized controlled trial (Li et al., 2018) recruited 22 AD patients and 8 healthy controls divided into MCI group, mild AD group, moderate–severe AD group and healthy controls. Frontal and parietal HbO concentrations were measured using fNIRS during a digit speech task, and the results of the study showed that abnormal haemodynamic responses were observed in the MCI group through to the moderate–severe AD group, with a greater decrease in HbO concentration with increasing disease severity. This test demonstrated that fNIRS detection of AD is feasible and also has great promise in exploring the mechanism of AD progression based on fNIRS. Olfactory impairment is one of the diagnostic criteria for MCI, and Kim et al. (2022) explored the potential diagnostic value of olfactory stimulation combined with fNIRS in the early detection of MCI and AD. The study included 28 healthy controls, 32 preclinical AD, 21 MCI, and 16 AD. All participants were tested for olfactory stimulation while receiving fNIRS to monitor the orbital frontal cortex. Orbital frontal cortex oxygenation was reduced in MCI and AD patients with olfactory stimulation compared to normal subjects, and this difference was positively correlated with MMSE isofunctional scores, i.e., cognitive deficits, and the level of orbital frontal cortex oxygenation did not differ from that of controls with non-olfactory stimulation. fNIRS in combination with olfactory stimulation may be a potential new diagnostic tool for patients with MCI and/or AD.

#### 4.2.3 Multimodal research applied to AD

Currently, both EEG and fNIRS have wide applications in AD research. eEG monitors EEG activity during cognitive training in AD patients and investigates the effects on cognitive function by observing changes in the characteristics of brain regions in different frequency bands (Abiri et al., 2019). fNIRS investigates the changes in cerebral blood flow and oxygenation levels and their effects on cognition and mood in AD patients during training (Acevedo et al., 2022). Hybrid EEG-fNIRS studies combining the complementary properties of both have better feasibility than EEG or fNIRS alone (Li et al., 2019). A study (Cicalese et al., 2020) explored the diagnostic value of combined EEG and fNIRS in an AD population, and concluded that the hybrid EEG-fNIRS model could be used in clinical treatment with higher accuracy for AD diagnosis. On this basis, Kim concluded that using EEG and fNIRS to measure brain signals individually or combining the two methods could monitor specific brain functions, but that Alzheimer’s disease research needed to monitor a variety of brain functions rather than focusing on a single one. Kim et al. (2023) proposed an innovative integrated research protocol for AD monitoring. The protocol integrates a 32-channel dry electrode EEG, a customized 4-channel fNIRS, and gait monitoring using a depth camera and pressure sensor. Different tasks target different brain functions. The protocol is expected to provide an easy-to-perform method of brain and gait monitoring for the elderly and AD patients.

Acupuncture therapy is a non-pharmacological treatment that is now commonly used for a variety of neurological disorders. However, fewer therapeutic studies of its effects on the neural mechanisms of AD patients have been reported in the literature. Ghafoor et al. (2019) innovatively used fNIRS to analyse the haemodynamic responses of patients after treatment with acupuncture therapy. The study showed that acupuncture therapy had a positive impact on improving cognitive function in AD patients. Acupuncture therapy may be used as a therapeutic tool for AD patients as a non-pharmacological treatment. It is expected that more literature will support the use of acupuncture in combination with fNIRS for AD treatment in the future.

#### 4.2.4 Summary

Herrmann et al. (2008) found that AD patients had a decrease in prefrontal lobe oxygenation during language fluency tasks, which was manifested by a decrease in HbR and an increase in HbO, but the increase in HbO was less in AD patients than in healthy controls. Arai et al. (2006) found that activation in the frontal and bilateral parietal areas in AD patients was significantly lower than that in the normal areas during the language fluency task, while in the MCI group it was significantly lower only in the right parietal area, confirming that the near-infrared spectroscopy system may be a potential tool for initial screening for Alzheimer’s disease. Metzger et al. (2016) found that during the language fluency task, the degree of cortical activation in the frontal and parietal lobe area increased in healthy controls, and the degree of activation also increased in AD patients, but not as much as in normal people. These studies confirm the reliability of fNIRS in AD.

Dysfunction of neurovascular units caused by aging, disease, etc. can lead to changes in neurovascular coupling (Vermeij et al., 2012). Over time, impaired neurovascular coupling can lead to tissue damage, loss of function, and cellular degradation. A large number of studies have shown that dysfunction of neurovascular units (NVU), which control neurovascular coupling mechanisms, is associated with various NDDs. Impaired cerebrovascular reactivity, dysregulation of reduced cerebral blood flow, and dysfunction of the blood–brain barrier are considered early events in the pathophysiological cascade of AD (Ferdinando et al., 2023).

Neural activity measured by fNRIS is positively correlated with blood oxygen level-dependent responses. fNIRS mainly identifies hemodynamic changes in early AD patients by discovering abnormalities in neurovascular coupling (Lee et al., 2024). At this time, AD patients may have no significant dysfunction or atrophy in the early stages of the disease. fNIRS only evaluates cortical changes, but cannot be detected in the hippocampus or entorhinal cortex, brain areas that play an important role in AD. It must be admitted that this greatly limits the development of fNIRS in AD (Zhang et al., 2023). Therefore, establishing the relationship between metabolic activity, structural changes, and amyloid plaque imaging detected by PET and MRI and the hemodynamic response detected by fNIRS can help assess the reliability of fNIRS. A study confirmed that the hemodynamic response in AD patients measured by fNIRS was correlated with metabolic and anatomical changes observed with PET (Yoon et al., 2023). But regarding the progress of AD,a direct relation of functional and structural data is not possible as fNIRS is only able to record functional activity and not structural information (Metzger et al., 2016). No conclusions can be drawn based on PET amyloid imaging and measurement of cortical thickness or hemodynamic data obtained through fNIRS (Ortner et al., 2019).

### 4.3 Amyotrophic lateral sclerosis

Amyotrophic lateral sclerosis (ALS) is a rare but serious neurodegenerative disease that leads to progressive loss of voluntary muscle control and a poor prognosis. In 2020, a clinical trial (Deligani et al., 2020) subjected 10 ALS and healthy controls to multimodal EEG-fNIRS monitoring in the resting state. Resting-state functional connectivity was compared and analysed between the two groups. The multimodal recordings presented in this study allow for a multidimensional study of functional network alterations under ALS pathology, and these results have the potential to be further extended as one of the tools for non-invasive diagnosis and prognosis of ALS.

#### 4.3.1 Multimodal research applied to ALS

Nowadays, the application of EEG in ALS related research is increasing gradually. Under the activation paradigm of EEG, event-related potential (ERP) reflects patients’ cognitive dysfunction, and P300 in ERP is currently an objective EEG biological index reflecting the degree of cognitive impairment (Raggi et al., 2010). The results of resting EEG studies preliminarily demonstrated the pattern of neuroelectrophysiological changes in the frontal, temporal and occipital lobes of the brain of ALS patients, and observed that the functional connectivity changes were correlated with cognitive function scores, suggesting that cognitive impairment in ALS involves extensive disruption of cognitive networks (Dukic et al., 2019). However, the spatial resolution and signal-to-noise ratio (SNR) of EEG are low, and for advanced ALS patients, the accuracy is affected by the lack of fine eye control due to the presence of ocular lesions, which makes the ERP-based paradigm assessment of ALS patients uncertain. One of the effective ways to solve this problem is to combine EEG with other neurofunctional imaging techniques to achieve complementarity. Given the progressive progression and later immobility of ALS patients, fNIRS measures brain hemodynamics and can be used directly at the patient’s bedside, making it a viable neuroimaging technique that complements EEG properties. It has been reported that analysis using EEG to measure ERP changes and fNIRS to measure HbR and HbO changes, as well as to assess the electrical (EEG) -blood flow (fNIRS) related mechanisms, reflects ALS non-motor function, and is expected to use these features as potential diagnostic and prognostic markers for ALS in the future (Borgheai et al., 2019).

The brain-computer interface (BCI) is a communication system that allows users to use brain activity to control a computer or other external device. It can provide a means of communication for people with severe movement disorders or persistent vegetative states, such as amyotrophic lateral sclerosis and spinal cord injuries (Webster, 2024). BCI systems require communication signal support. fNIRS is a novel BCI signal. Coyle et al. (2004) first verified the feasibility of applying fNIRS to BCI. Hong et al. measured and distinguished fNIRS signals evoked by three different mental activities, namely mental arithmetic (MA), right-hand motor imagination (RI), and left-hand motor imagination (LI). Ten healthy subjects were asked to complete MA, RI and LI over a 10-s task period while simultaneously receiving signals from the prefrontal lobe and primary motor cortex. The feasibility of using three different cognitive tasks as inputs to implement fNIRS-BCI was demonstrated (Hong et al., 2015). Hosni et al. (2020) explored the specific spatio-temporal characteristics of the hemodynamic responses of ALS patients to motion-based image-based tasks, recorded the hemodynamic responses of eight ALS patients, emphasizing the importance of subject-specific data-driven methods in identifying discriminative spatio-temporal characteristics to optimize BCI performance. Borgheai et al. (2020) explored the feasibility of a fNIRS based BCI system to enhance communication in ALS patients, especially in the later stages of the disease. Studies have confirmed the potential efficacy of the system in communication in patients with advanced ALS.

### 4.4 Multiple sclerosis

Multiple sclerosis (Marcus, 2022) (MS) is a neurodegenerative disease characterized by chronic inflammatory demyelination of the central nervous system. Its main clinical manifestations are limb weakness, ataxia, eye discomfort and mental symptoms. In the past, the diagnosis of MS mainly depended on clinical manifestations and pathological examination. In recent years, fNIRS has been used in the early detection of MS patients. Patients with MS have gait disorder and cognitive impairment when performing dual tasks. Stojanovic-Radic et al. (2015) used the medium fNIRS method to study brain activation during cognitive tasks in MS patients for the first time. The study included 13 MS patients and 12 healthy controls and recorded fNIRS during tasks at four difficulty levels. Studies have shown that brain activation patterns are different in MS patients and healthy controls. The oxygenated hemoglobin concentration in the left superior frontal gyrus of the MS group showed an increase in brain activation at a lower task difficulty level, while in a higher task difficulty level, the brain activation of the MS group decreased. The study shows that fNIRS is very sensitive to the changes of HbO and HbR and can detect neuronal activation in detail, which provides an inspiration for the follow-up fNIRS technology to detect MS. Hernandez et al. (2016) used fNIRS to study the changes in brain activation in patients with MS during simple and distracting motor tasks. 8 patients with MS and 8 healthy controls were recorded by fNIRS while performing dual tasks (talking and walking). Compared with healthy controls, the level of PFC oxygenation in MS group was higher during dual task. During the whole exercise, there was no difference in walking ability among different groups, suggesting that patients with MS may achieve similar performance levels in healthy controls by increasing brain activation compensation. On this basis, de Aratanha et al. (2022) used fNIRS and gait parameters to evaluate cortical hemodynamics in early MS patients and healthy controls under dual tasks. Participants performed cognitive tasks to simulate daily activities while walking, and cortical activation maps and gait variability were used to assess the differences between 19 healthy controls and 20 MS patients. The results showed that cortical activation in the exercise planning area was increased in the early stages of MS compared with the control group. The correlation between cortical and gait parameters may reveal the possible compensatory mechanism related to gait during dual tasks in the early stage of the disease. At present, there are few literatures on the application of fNIRS to MS, and it is still in its infancy. We look forward to a strong breakthrough in the application of fNIRS in MS in the future.

### 4.5 Other diseases

#### 4.5.1 Multiple system atrophy

Multiple system atrophy (MSA) is a group of disseminated NDDs of unknown etiology involving the extrapyramidal system, pyramidal system, cerebellum, and autonomic nervous system, with a rapid progression of the disease and a lack of effective treatments, and is characterized by early onset of autonomic dysfunction (Poewe et al., 2022). A study (Suzuki et al., 2008) used fNIRS to investigate the feasibility of brain tissue oximetry monitoring in assessing cerebral circulation in patients with MSA. It was found that the cerebral oxygenation response assessed by fNIRS reflected arterial, venous and tissue oxygen saturation and was closely related to cerebral blood flow. fNIRS is useful in detecting changes in cerebral oxygenation associated with cerebral hypoperfusion and may be useful in detecting the lower limit of cerebral autoregulation in patients with MSA.

#### 4.5.2 Frontotemporal lobe dementia

Frontotemporal lobe dementia (FTD) (Olney et al., 2017) is a type of NDDs with severe cognitive and behavioral deficits due to frontotemporal lobe degeneration, which is not easy to be detected and has an early onset, and most often manifests progressive worsening of behavioral abnormality, language impairment, and social cognitive deficits, etc.; Dementia with Lewy bodies (DLB) is a type of neurodegenerative dementia due to abnormal aggregation of Lewy bodies and *α*-synuclein, and is the second most common cognitive disorder after AD. In 2023, a study (Mei et al., 2023) examined four different types of dementia, PD, AD, FTD, and DLB, using fNIRS, in patients with PD in both task and resting states. The study have found that FTD patients present with decreased or atrophied frontotemporal lobe function, which may be accompanied by low levels of decreased frontotemporal oxygen-hemoglobin function. AD patients had lower and slower activation of the bilateral PFC and left parietal cortex during working memory maintenance; PD patients had higher excitability of the prefrontal cortex during the working memory task, with strong activation of the dorsolateral prefrontal cortex; and LBD patients showed a strong asymmetry between the two hemispheres with severe asymmetry, and patients had lower strength of functional connectivity in the resting state. The different haemodynamic profiles of the four types of dementia provide evidence that fNIRS can be used as a potential tool for diagnosing between subtypes of dementia.

## 5 Discussion

Unlike other neuroimaging technologies, fNIRS has an irreplaceable position in the field of NDDs due to its unique neurooxygenation signal brain function imaging technology features to monitor the condition, formulate the treatment plan, and evaluate the treatment effect. fNIRS also has the advantages of easy operation, low cost, high safety, non-invasiveness, good compatibility, and high temporal resolution, and has been widely used in the diseases of PD, AD, and stroke. However, given the limitations of its technology, fNIRS cannot replace other imaging examinations and invasive monitoring at present, and has a broad application prospect with insufficient clinical studies in certain NDDs. In future research, fNIRS can be used in conjunction with EEG, tDCS and other technologies as a multimodal brain function examination to achieve technological complementation and conduct deeper research on the brain. fNIRS can also be used as an important supplement to imaging examinations to improve anatomical localisation accuracy and provide personalized treatment, highlighting the value of the application of fNIRS in the prediction of diagnosis of NDDs and better utilize the unique advantages of fNIRS.

## References

[ref1] Abiri R. Borhani S. Sellers E. W. Jiang Y. Zhao X. (2019). A comprehensive review of EEG-based brain-computer interface paradigms. J. Neural Eng. 16:011001. doi: 10.1088/1741-2552/aaf12e, PMID: 30523919

[ref2] Abtahi M. Bahram Borgheai S. Jafari R. Constant N. Diouf R. Shahriari Y.. (2020). Merging fNIRS-EEG brain monitoring and body motion capture to distinguish Parkinsons disease. IEEE Trans. Neural Syst. Rehabil. Eng. 28, 1246–1253. doi: 10.1109/TNSRE.2020.2987888, PMID: 32305929

[ref3] Acevedo B. P. Dattatri N. Le J. Lappinga C. Collins N. L. (2022). Cognitive training with Neurofeedback using fNIRS improves cognitive function in older adults. Int. J. Environ. Res. Public Health 19:5531. doi: 10.3390/ijerph19095531, PMID: 35564926 PMC9104766

[ref4] Arai H. Takano M. Miyakawa K. Ota T. Takahashi T. Asaka H.. (2006). A quantitative near-infrared spectroscopy study: a decrease in cerebral hemoglobin oxygenation in Alzheimer's disease and mild cognitive impairment. Brain Cogn. 61, 189–194. doi: 10.1016/j.bandc.2005.12.012 16466836

[ref5] Balakrishnan B. Dasgupta M. Gajewski K. Hoffmann R. G. Simpson P. M. Havens P. L.. (2018). Low near infrared spectroscopic somatic oxygen saturation at admission is associated with need for lifesaving interventions among unplanned admissions to the pediatric intensive care unit. J. Clin. Monit. Comput. 32, 89–96. doi: 10.1007/s10877-017-0007-1, PMID: 28258341

[ref6] Borgheai S. B. Deligani R. J. McLinden J. Zisk A. Hosni S. I. Abtahi M.. (2019). Multimodal exploration of non-motor neural functions in ALS patients using simultaneous EEG-fNIRS recording. J. Neural Eng. 16:066036. doi: 10.1088/1741-2552/ab456c, PMID: 31530755 PMC7093150

[ref7] Borgheai S. B. McLinden J. Zisk A. H. Hosni S. I. Deligani R. J. Abtahi M.. (2020). Enhancing communication for people in late-stage ALS using an fNIRS-based BCI system. IEEE Trans. Neural Syst. Rehabil. Eng. 28, 1198–1207. doi: 10.1109/TNSRE.2020.2980772, PMID: 32175867 PMC7288752

[ref8] Cicalese P. A. Li R. Ahmadi M. B. Wang C. Francis J. T. Selvaraj S.. (2020). An EEG-fNIRS hybridization technique in the four-class classification of Alzheimer’s disease. J. Neurosci. Methods 336:108618. doi: 10.1016/j.jneumeth.2020.108618 32045572 PMC7376762

[ref9] Conceição N. R. Gobbi L. T. B. Nóbrega-Sousa P. Orcioli-Silva D. Beretta V. S. Lirani-Silva E.. (2021). Aerobic exercise combined with transcranial direct current stimulation over the prefrontal cortex in Parkinson disease: effects on cortical activity, gait, and cognition. Neurorehabil. Neural Repair 35, 717–728. doi: 10.1177/15459683211019344, PMID: 34047235

[ref10] Coyle S. Ward T. Markham C. McDarby G. (2004). On the suitability of near-infrared (NIR) systems for next-generation brain-computer interfaces. Physiol. Meas. 25, 815–822. doi: 10.1088/0967-3334/25/4/003, PMID: 15382823

[ref11] Criado J. M. de la Fuente A. Heredia M. Riolobos A. S. Yajeya J. (1997). Electrophysiological study of prefrontal neurones of cats during a motor task. Pflugers Arch. 434, 91–96. doi: 10.1007/s004240050367, PMID: 9094260

[ref12] Curtin A. Tong S. Sun J. Wang J. Onaral B. Ayaz H. (2019). A systematic review of integrated functional near-infrared spectroscopy(fNIRS)and transcranial magnetic stImulation (TMS) studies. Front. Neurosci. 13:84. doi: 10.3389/fnins.2019.00084, PMID: 30872985 PMC6403189

[ref13] de Aratanha M. A. Balardin J. B. Cardoso do Amaral C. Lacerda S. S. Sowmy T. A. S. Huppert T. J.. (2022). The use of functional near infrared spectroscopy and gait analysis to characterize cognitive and motor processing in early-stage patients with multiple sclerosis. Front. Neurol. 13:937231. doi: 10.3389/fneur.2022.937231, PMID: 36105774 PMC9464830

[ref14] de Hemptinne C. Swann N. C. Ostrem J. L. Ryapolova-Webb E. S. San Luciano M. Galifianakis N. B.. (2015). Therapeutic deep brain stimulation reduces cortical phase-amplitude coupling in Parkinson's disease. Nat. Neurosci. 18, 779–786. doi: 10.1038/nn.3997, PMID: 25867121 PMC4414895

[ref15] Deligani R. J. Hosni S. I. Borgheai S. B. McLinden J. Zisk A. H. Mankodiya K.. (2020). Electrical and hemodynamic neural functions in people with ALS: an EEG-fNIRS resting-state study. IEEE Trans. Neural Syst. Rehabil. Eng. 28, 3129–3139. doi: 10.1109/TNSRE.2020.3031495, PMID: 33055020 PMC7952040

[ref16] Dukic S. McMackin R. Buxo T. Fasano A. Chipika R. Pinto-Grau M.. (2019). Patterned functional network disruption in amyotrophic lateral sclerosis. Hum. Brain Mapp. 40, 4827–4842. doi: 10.1002/hbm.24740, PMID: 31348605 PMC6852475

[ref17] Ferdinando H. Moradi S. Korhonen V. Kiviniemi V. Myllylä T. (2023). Altered cerebrovascular-CSF coupling in Alzheimer's disease measured by functional near-infrared spectroscopy. Sci. Rep. 13:22364. doi: 10.1038/s41598-023-48965-x, PMID: 38102188 PMC10724150

[ref18] Ferrari M. Quaresima V. (2012). A brief review on the history of human functional near-infrared spectroscopy (fNIRS) development and fields of application. NeuroImage 63, 921–935. doi: 10.1016/j.neuroimage.2012.03.049, PMID: 22510258

[ref19] Fransson P. Skiöld B. Engström M. Hallberg B. Mosskin M. Aden U.. (2009). Spontaneous brain activity in the newborn brain during natural sleep--an fMRI study in infants born at full term. Pediatr. Res. 66, 301–305. doi: 10.1203/PDR.0b013e3181b1bd84, PMID: 19531974

[ref20] Ghafoor U. Lee J. H. Hong K. S. Park S. S. Kim J. Yoo H. R. (2019). Effects of acupuncture therapy on MCI patients using functional near-infrared spectroscopy. Front. Aging Neurosci. 11:237. doi: 10.3389/fnagi.2019.00237, PMID: 31543811 PMC6730485

[ref21] Gustavsson A. Norton N. Fast T. Frölich L. Georges J. Holzapfel D.. (2023). Global estimates on the number of persons across the Alzheimer's disease continuum. Alzheimers Dement. 19, 658–670. doi: 10.1002/alz.12694 35652476

[ref22] Hamacher D., Herold F. Wiegel P. Hamacher D. Schega L. Brain activity during walking: a systematic review. Neurosci. Biobehav. Rev. (2015). 57, 310–327. doi: 10.1016/j.neubiorev.2015.08.002, PMID: 26306029

[ref23] Hernandez M. E. Holtzer R. Chaparro G. Jean K. Balto J. M. Sandroff B. M.. (2016). Brain activation changes during locomotion in middle-aged to older adults with multiple sclerosis. J. Neurol. Sci. 370, 277–283. doi: 10.1016/j.jns.2016.10.002, PMID: 27772776

[ref24] Herrmann M. J. Langer J. B. Jacob C. Ehlis A. C. Fallgatter A. J. (2008). Reduced prefrontal oxygenation in Alzheimer disease during verbal fluency tasks. Am. J. Geriatr. Psychiatry 16, 125–135. doi: 10.1097/JGP.0b013e3180cc1fbc, PMID: 17998307

[ref25] Hock C. Villringer K. Müller-Spahn F. Hofmann M. Schuh-Hofer S. Heekeren H.. (1996). Near infrared spectroscopy in the diagnosis of Alzheimer's disease. Ann. N. Y. Acad. Sci. 777, 22–29. doi: 10.1111/j.1749-6632.1996.tb34397.x 8624087

[ref26] Hoffman G. M. Ghanayem N. S. Scott J. P. Tweddell J. S. Mitchell M. E. Mussatto K. A. (2017). Postoperative cerebral and somatic near-infrared spectroscopy saturations and outcome in hypoplastic left heart syndrome. Ann. Thorac. Surg. 103, 1527–1535. doi: 10.1016/j.athoracsur.2016.09.100, PMID: 28012642

[ref27] Hong K. S. Naseer N. Kim Y. H. (2015). Classification of prefrontal and motor cortex signals for three-class fNIRS-BCI. Neurosci. Lett. 587, 87–92. doi: 10.1016/j.neulet.2014.12.029, PMID: 25529197

[ref28] Hosni S. M. Borgheai S. B. McLinden J. Shahriari Y. (2020). An fNIRS-based motor imagery BCI for ALS: a subject-specific data-driven approach. IEEE Trans. Neural Syst. Rehabil. Eng. 28, 3063–3073. doi: 10.1109/TNSRE.2020.3038717, PMID: 33206606

[ref29] Iadecola C. (2017). The neurovascular unit coming of age: a journey through neurovascular coupling in health and disease. Neuron 96, 17–42. doi: 10.1016/j.neuron.2017.07.030, PMID: 28957666 PMC5657612

[ref30] Irani F. Platek S. M. Bunce S. Ruocco A. C. Chute D. (2007). Functional near infrared spectroscopy (fNIRS): an emerging neuroimaging technology with important applications for the study of brain disorders. Clin. Neuropsychol. 21, 9–37. doi: 10.1080/13854040600910018, PMID: 17366276

[ref31] Jalakas M. Palmqvist S. Hall S. Svärd D. Lindberg O. Pereira J. B.. (2019). A quick test of cognitive speed can predict development of dementia in Parkinson's disease. Sci. Rep. 9:15417. doi: 10.1038/s41598-019-51505-1, PMID: 31659172 PMC6817840

[ref32] Jasdzewski G. Strangman G. Wagner J. Kwong K. K. Poldrack R. A. Boas D. A. (2003). Differences in the hemodynamic response to event-related motor and visual paradigms as measured by near-infrared spectroscopy. NeuroImage 20, 479–488. doi: 10.1016/S1053-8119(03)00311-2, PMID: 14527608

[ref33] Jöbsis F. F. (1977). Noninvasive, infrared monitoring of cerebral and myocardial oxygen sufficiency and circulatory parameters. Science 198, 1264–1267. doi: 10.1126/science.929199, PMID: 929199

[ref34] Keles H. O. Barbour R. L. Omurtag A. (2016). Hemodynamic correlates of spontaneous neural activity measured by human whole-head resting state EEG+fNIRS. NeuroImage 138, 76–87. doi: 10.1016/j.neuroimage.2016.05.058, PMID: 27236081

[ref35] Kim J. Yon D. K. Choi K. Y. Lee J. J. Kim N. Lee K. H.. (2022). Novel diagnostic tools for identifying cognitive impairment using olfactory-stimulated functional near-infrared spectroscopy: patient-level, single-group, diagnostic trial. Alzheimers Res. Ther. 14:39. doi: 10.1186/s13195-022-00978-w 35260170 PMC8905807

[ref36] Kim M. Jang S. Lee D. Lee S. Gwak J. Jun S. C.. (2023). A comprehensive research setup for monitoring Alzheimer's disease using EEG, fNIRS, and gait analysis. Biomed. Eng. Lett. 14, 13–21. doi: 10.1007/s13534-023-00306-7, PMID: 38186957 PMC10769970

[ref37] Koenraadt K. L. Roelofsen E. G. Duysens J. Keijsers N. L. (2014). Cortical control of normal gait and precision stepping: an fNIRS study. NeuroImage 85, 415–422. doi: 10.1016/j.neuroimage.2013.04.070, PMID: 23631980

[ref38] Lee T. L. Guo L. Chan A. S. (2024). fNIRS as a biomarker for individuals with subjective memory complaints and MCI. Alzheimers Dement. 20, 5170–5182. doi: 10.1002/alz.13897, PMID: 38837656 PMC11350052

[ref39] Li R. Nguyen T. Potter T. Zhang Y. (2019). Dynamic cortical connectivity alterations associated with Alzheimer's disease: an EEG and fNIRS integration study. Neuroimage Clin. 21:101622. doi: 10.1016/j.nicl.2018.101622, PMID: 30527906 PMC6411655

[ref40] Li R. Rui G. Chen W. Li S. Schulz P. E. Zhang Y. (2018). Early detection of Alzheimer's disease using non-invasive near-infrared spectroscopy. Front. Aging Neurosci. 10:366. doi: 10.3389/fnagi.2018.00366 30473662 PMC6237862

[ref41] Lim S. B. Yang C. L. Peters S. Liu-Ambrose T. Boyd L. A. Eng J. J. (2022). Phase-dependent brain activation of the frontal and parietal regions during walking after stroke - an fNIRS study. Front. Neurol. 13:904722. doi: 10.3389/fneur.2022.904722, PMID: 35928123 PMC9343616

[ref42] Logroscino G. Urso D. Savica R. (2022). Descriptive epidemiology of neurodegenerative diseases: what are the critical questions? Neuroepidemiology 56, 309–318. doi: 10.1159/000525639, PMID: 35728570 PMC9677839

[ref43] Mahoney J. R. Holtzer R. Izzetoglu M. Zemon V. Verghese J. Allali G. (2016). The role of prefrontal cortex during postural control in parkinsonian syndromes a functional near-infrared spectroscopy study. Brain Res. 1633, 126–138. doi: 10.1016/j.brainres.2015.10.053, PMID: 26551767 PMC4860007

[ref44] Maidan I. Nieuwhof F. Bernad-Elazari H. Reelick M. F. Bloem B. R. Giladi N.. (2016). The role of the frontal lobe in complex walking among patients with Parkinson's disease and healthy older adults: an fNIRS study. Neurorehabil. Neural Repair 30, 963–971. doi: 10.1177/1545968316650426, PMID: 27221042

[ref45] Marcus R. (2022). What is multiple sclerosis? JAMA 328:2078. doi: 10.1001/jama.2022.14236 36413229

[ref46] Mei X. Zou C.-J. Hu J. Liu X.-L. Zheng C.-Y. Zhou D.-S. (2023). Functional near-infrared spectroscopy in elderly patients with four types of dementia. World J. Psychiatry 13, 203–214. doi: 10.5498/wjp.v13.i5.203 37303929 PMC10251357

[ref47] Metzger F. G. Schopp B. Haeussinger F. B. Dehnen K. Synofzik M. Fallgatter A. J.. (2016). Brain activation in frontotemporal and Alzheimer's dementia: a functional near-infrared spectroscopy study. Alzheimers Res. Ther. 8:56. doi: 10.1186/s13195-016-0224-8, PMID: 27931245 PMC5146884

[ref48] Mirelman A. Bonato P. Camicioli R. Ellis T. D. Giladi N. Hamilton J. L.. (2019). Gait impairments in Parkinson's disease. Lancet Neurol. 18, 697–708. doi: 10.1016/S1474-4422(19)30044-4 30975519

[ref49] Morris H. R. Spillantini M. G. Sue C. M. Williams-Gray C. H. (2024). The pathogenesis of Parkinson's disease. Lancet 403, 293–304. doi: 10.1016/S0140-6736(23)01478-2 38245249

[ref50] Nippert A. R. Biesecker K. R. Newman E. A. (2018). Mechanisms mediating functional hyperemia in the brain. Neuroscientist 24, 73–83. doi: 10.1177/1073858417703033 28403673 PMC5757525

[ref51] Noah J. A. Ono Y. Nomoto Y. Shimada S. Tachibana A. Zhang X.. (2015). fMRI validation of fNIRS measurements during a naturalistic task. J. Vis. Exp. 100:e52116. doi: 10.3791/52116-v PMC4544944 26132365

[ref52] Olney N. T. Spina S. Miller B. L. (2017). Frontotemporal Dementia. Neurol. Clin. 35, 339–374. doi: 10.1016/j.ncl.2017.01.008, PMID: 28410663 PMC5472209

[ref53] Orcioli-Silva D. Vitório R. Nóbrega-Sousa P. Beretta V. S. Conceição N. R. D. Oliveira A. S.. (2021). Cortical activity underlying gait improvements achieved with dopaminergic medication during usual walking and obstacle avoidance in Parkinson disease. Neurorehabil. Neural Repair 35, 406–418. doi: 10.1177/15459683211000736 33754884

[ref54] Ortner M. Drost R. Heddderich D. Goldhardt O. Müller-Sarnowski F. Diehl-Schmid J.. (2019). Amyloid PET, FDG-PET or MRI?-the power of different imaging biomarkers to detect progression of early Alzheimer’s disease. BMC Neurol. 19, 1–6. doi: 10.1186/s12883-019-1498-9 31672138 PMC6822351

[ref55] Pasko V. I. Churkina A. S. Shakhov A. S. Kotlobay A. A. Alieva I. B. (2022). Modeling of neurodegenerative diseases: 'Step by Step' and 'Network' Organization of the complexes of model systems. Int. J. Mol. Sci. 24:604. doi: 10.3390/ijms24010604, PMID: 36614047 PMC9820769

[ref56] Patel R. Dawidziuk A. Darzi A. Singh H. Leff D. R. (2020). Systematic review of combined functional near-infrared spectroscopy and transcranial direct-current stimulation studies. Neurophotonics. 7:020901. doi: 10.1117/1.NPh.7.2.020901, PMID: 32607389 PMC7315225

[ref57] Pinti P. Tachtsidis I. Hamilton A. Hirsch J. Aichelburg C. Gilbert S.. (2020). The present and future use of functional near-infrared spectroscopy (fNIRS) for cognitive neuroscience. Ann. N. Y. Acad. Sci. 1464, 5–29. doi: 10.1111/nyas.13948, PMID: 30085354 PMC6367070

[ref58] Poewe W. Stankovic I. Halliday G. Meissner W. G. Wenning G. K. Pellecchia M. T.. (2022). Multiple system atrophy. Nat. Rev. Dis. Primers 8:56. doi: 10.1038/s41572-022-00382-6 36008429

[ref59] Rabin L. A. Smart C. M. Amariglio R. E. (2017). Subjective cognitive decline in preclinical Alzheimer's disease. Annu. Rev. Clin. Psychol. 13, 369–396. doi: 10.1146/annurev-clinpsy-032816-045136 28482688

[ref60] Raggi A. Iannaccone S. Cappa S. F. (2010). Event-related brain potentials in amyotrophic lateral sclerosis: a review of the international literature. Amyotroph. Lateral Scler. 11, 16–26. doi: 10.3109/17482960902912399, PMID: 19412819

[ref61] Rahman M. A. Siddik A. B. Ghosh T. K. Khanam F. Ahmad M. (2020). A narrative review on clinical applications of fNIRS. J. Digit. Imaging 33, 1167–1184. doi: 10.1007/s10278-020-00387-1, PMID: 32989620 PMC7573058

[ref62] San Juan J. Hu X. S. Issa M. Bisconti S. Kovelman I. Kileny P.. (2017). Tinnitus alters resting state functional connectivity(RSFC)in human auditory and non-auditory brain regions as measured by functional near-infrared spectroscopy(fNIRS). PLoS One 12:e0179150. doi: 10.1371/journal.pone.0179150, PMID: 28604786 PMC5467838

[ref63] Schejter-Margalit T. Kizony R. Ben-Binyamin N. Hacham R. Thaler A. Maidan I.. (2022). Neural activation in the prefrontal cortex during the digital clock drawing test measured with functional near-infrared spectroscopy in early stage Parkinson's disease. Parkinsonism Relat. Disord. 105, 9–14. doi: 10.1016/j.parkreldis.2022.10.021, PMID: 36327601

[ref64] Scheltens P. De Strooper B. Kivipelto M. Holstege H. Chételat G. Teunissen C. E.. (2021). Alzheimer's disease. Lancet 397, 1577–1590. doi: 10.1016/S0140-6736(20)32205-4, PMID: 33667416 PMC8354300

[ref65] Scholkmann F. Kleiser S. Metz A. J. Zimmermann R. Mata Pavia J. Wolf U.. (2014). A review on continuous wave functional near-infrared spectroscopy and imaging instrumentation and methodology. NeuroImage 85, 6–27. doi: 10.1016/j.neuroimage.2013.05.004, PMID: 23684868

[ref66] Stojanovic-Radic J. Wylie G. Voelbel G. Chiaravalloti N. DeLuca J. (2015). Neuroimaging and cognition using functional near infrared spectroscopy (fNIRS) in multiple sclerosis. Brain Imaging Behav. 9, 302–311. doi: 10.1007/s11682-014-9307-y, PMID: 24916919

[ref67] Strangman G. Boas D. A. Sutton J. P. (2002). Non-invasive neuroimaging using near-infrared light. Biol. Psychiatry 52, 679–693. doi: 10.1016/S0006-3223(02)01550-0 12372658

[ref68] Suzuki K. Asahina M. Suzuki A. Hattori T. (2008). Cerebral oxygenation monitoring for detecting critical cerebral hypoperfusion in patients with multiple system atrophy during the head-up tilt test. Intern. Med. 47, 1681–1687. doi: 10.2169/internalmedicine.47.1094, PMID: 18827416

[ref69] Uchitel J. Vidal-Rosas E. E. Cooper R. J. Zhao H. (2021). Wearable, integrated EEG-fNIRS technologies: a review. Sensors 21:6106. doi: 10.3390/s21186106, PMID: 34577313 PMC8469799

[ref70] Vermeij A. van Beek A. H. Olde Rikkert M. G. Claassen J. A. Kessels R. P. (2012). Effects of aging on cerebral oxygenation during working-memory performance: a functional near-infrared spectroscopy study. PLoS One 7:e46210. doi: 10.1371/journal.pone.0046210, PMID: 23029437 PMC3460859

[ref71] Vitek J. L. Starr P. A. (2020). Studies of deep brain stimulation in Parkinson's disease. Lancet Neurol. 19, 807–808. doi: 10.1016/S1474-4422(20)30323-9 32949537

[ref72] Webster P. (2024). The future of brain-computer interfaces in medicine. Nat. Med. 30, 1508–1509. doi: 10.1038/d41591-024-00031-3, PMID: 38714865

[ref73] Weller S. Nitsche M. A. Plewnia C. (2020). Enhancing cognitive control training with transcranial direct current stimulation: a systematic parameter study. Brain Stimul. 13, 1358–1369. doi: 10.1016/j.brs.2020.07.006, PMID: 32687899

[ref74] Xu Y. McClelland V. M. Cvetkovic Z. Mills K. R. (2017). Corticomuscular coherence with time lag with application to delay estimation. I.E.E.E. Trans. Biomed. Eng. 64, 588–600. doi: 10.1109/TBME.2016.2569492, PMID: 27214885

[ref75] Yoon J. A. Kong I. J. Choi I. Cha J. Baek J. Y. Choi J.. (2023). Correlation between cerebral hemodynamic functional near-infrared spectroscopy and positron emission tomography for assessing mild cognitive impairment and Alzheimer's disease: an exploratory study. PLoS One 18:e0285013. doi: 10.1371/journal.pone.0285013, PMID: 37561711 PMC10414577

[ref76] Yu N. Liang S. Lu J. Shu Z. Li H. Yu Y.. (2021). Quantified assessment of deep brain stimulation on Parkinson's patients with task fNIRS measurements and functional connectivity analysis: a pilot study. Chin Neurosurg J. 7:34. doi: 10.1186/s41016-021-00251-3 34225815 PMC8256573

[ref77] Zhang Y. Zhang Y. Jiang Z. Xu M. Qing K. (2023). The effect of EEG and fNIRS in the digital assessment and digital therapy of Alzheimer's disease: a systematic review. Front. Neurosci. 17:1269359. doi: 10.3389/fnins.2023.1269359, PMID: 38075282 PMC10703187

[ref78] Zheng Y. Gao L. Wang G. Wang Y. Yang Z. Wang X.. (2016). The influence of unilateral contraction of hand muscles on the contralateral corticomuscular coherence during bimanual motor tasks. Neuropsychologia 85, 199–207. doi: 10.1016/j.neuropsychologia.2016.03.028, PMID: 27018484

[ref79] Zhou Z. D. Yi L. X. Wang D. Q. Lim T. M. Tan E. K. (2023). Role of dopamine in the pathophysiology of Parkinson's disease. Transl Neurodegener. 12:44. doi: 10.1186/s40035-023-00378-6, PMID: 37718439 PMC10506345

